# Supplementary material for: The causal relationship between the human gut microbiota and pyogenic arthritis: a Mendelian randomization study
Source: Front Cell Infect Microbiol. 2024 Nov 26;14:1452480. doi: 10.3389/fcimb.2024.1452480 (PMC11629706; doi:10.3389/fcimb.2024.1452480)
Supplement: Supplementary file 1 [file DataSheet1.zip › mendelian test/ebi-a-GCST90027844.csv_leave_one_out.pdf]

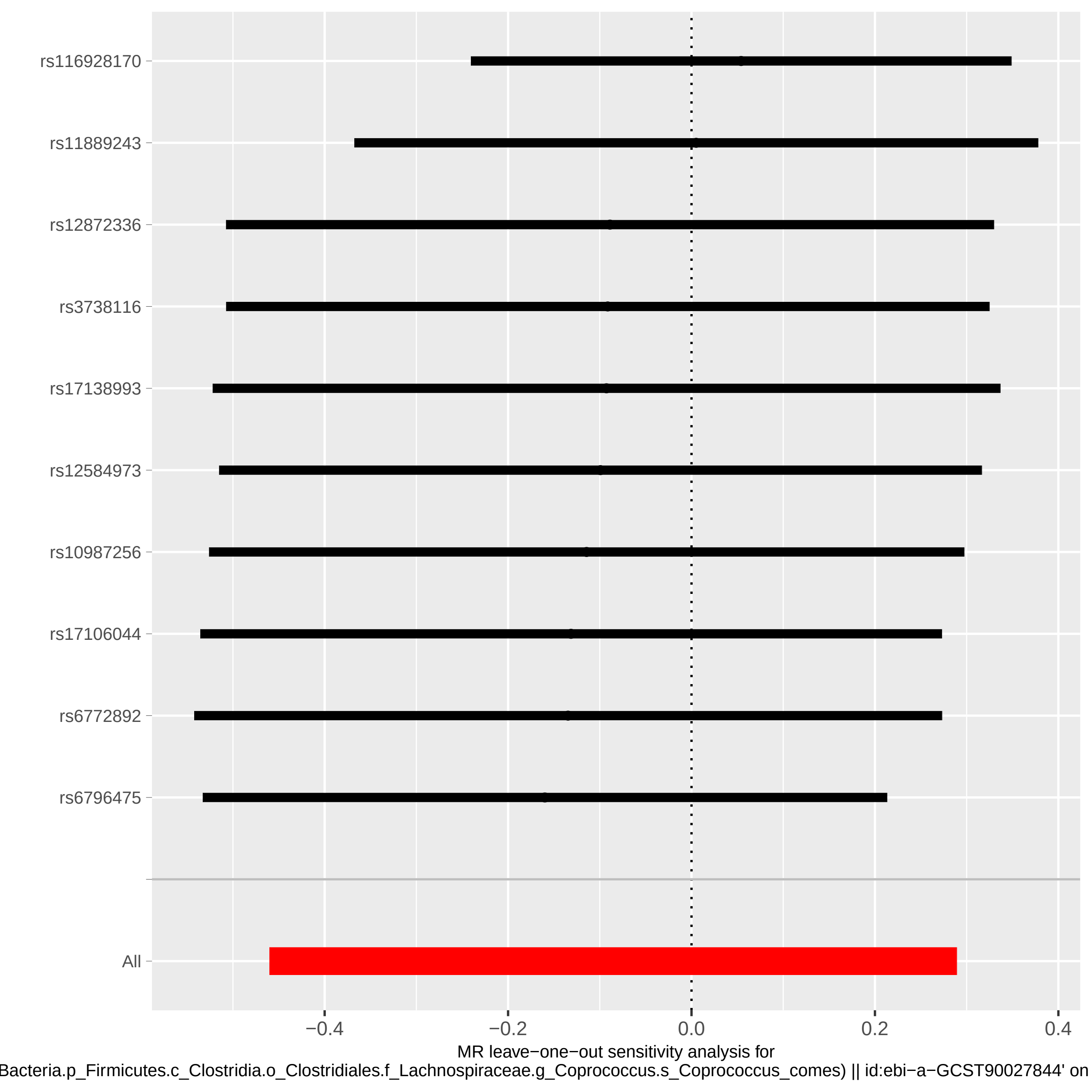
rs116928170
rs11889243
rs12872336
rs3738116
rs17138993
rs12584973
rs10987256
rs17106044
rs6772892
rs6796475
All
−0.4
−0.2
0.0
0.2
0.4
MR leave−one−out sensitivity analysis for
Bacteria.p_Firmicutes.c_Clostridia.o_Clostridiales.f_Lachnospiraceae.g_Coprococcus.s_Coprococcus_comes) || id:ebi−a−GCST90027844' on
